# Forecasting Strength of CFRP Confined Concrete Using Multi Expression Programming

**DOI:** 10.3390/ma14237134

**Published:** 2021-11-24

**Authors:** Israr Ilyas, Adeel Zafar, Muhammad Faisal Javed, Furqan Farooq, Fahid Aslam, Muhammad Ali Musarat, Nikolai Ivanovich Vatin

**Affiliations:** 1 Department of Structural Engineering, Military College of Engineering (MCE), National University of Science and Technology (NUST), Islamabad 44000, Pakistan; israr.awaan@gmail.com (I.I.); adeel.zafar@mce.nust.edu.pk (A.Z.); 2 Department of Civil Engineering, COMSATS University Islamabad, Abbottabad Campus, Abbottabad 22060, Pakistan; 3 Faculty of Civil Engineering, Cracow University of Technology, 24 Warszawska Str., 31-155 Cracow, Poland; furqan@cuiatd.edu.pk; 4 Department of Civil Engineering, College of Engineering in Al-Kharj, Prince Sattam bin Abdulaziz University, Al-Kharj 11942, Saudi Arabia; f.aslam@psau.edu.sa; 5 Department of Civil and Environmental Engineering, Bandar Seri Iskandar 32610, Perak, Malaysia; muhammad_19000316@utp.edu.my; 6 Peter the Great St. Petersburg Polytechnic University, 195291 St. Petersburg, Russia; vatin@mail.ru

**Keywords:** multiphysics model, multi expression programming, carbon fiber-reinforced polymer, parametric analysis, prediction

## Abstract

This study provides the application of a machine learning-based algorithm approach names “Multi Expression Programming” (MEP) to forecast the compressive strength of carbon fiber-reinforced polymer (CFRP) confined concrete. The suggested computational Multiphysics model is based on previously reported experimental results. However, critical parameters comprise both the geometrical and mechanical properties, including the height and diameter of the specimen, the modulus of elasticity of CFRP, unconfined strength of concrete, and CFRP overall layer thickness. A detailed statistical analysis is done to evaluate the model performance. Then the validation of the soft computational model is made by drawing a comparison with experimental results and other external validation criteria. Moreover, the results and predictions of the presented soft computing model are verified by incorporating a parametric analysis, and the reliability of the model is compared with available models in the literature by an experimental versus theoretical comparison. Based on the findings, the valuation and performance of the proposed model is assessed with other strength models provided in the literature using the collated database. Thus the proposed model outperformed other existing models in term of accuracy and predictability. Both parametric and statistical analysis demonstrate that the proposed model is well trained to efficiently forecast strength of CFRP wrapped structural members. The presented study will promote its utilization in rehabilitation and retrofitting and contribute towards sustainable construction material.

## 1. Introduction

Compared to steel and other retrofitting techniques, carbon fiber-reinforced polymer (CFRP) possesses significant properties such as high tensile strength, resistance towards the corrosive environment, minimal maintenance, improved aesthetics, reduced thermal and electrical conductivity, strong resistance to chemical assaults, stress durability, and geometric compatibility. Thus, due to the exceptional CFRP’s characteristics, they have been utilized as exterior confinement to improve further concrete components’ compressive and flexural capacity when the core reinforcement is inadequate to hold the stresses. However, when earthquakes inflict damage to concrete structures, their strength and serviceability deteriorate, and their restoration requires retrofitting or rehabilitation through different techniques. In this regard, jacketing through CFRP is also considered dominant in contrast to steel and concrete jacketing due to many aspects, including convenient handling and installation, minimal disruption of structure, and reduced time utilization. Moreover, CFRPs being popular in other domains can also be incorporated for retrofitting and rehabilitation of buildings and bridges to enhance the strength and efficacy of structures [1,2,3,4,5,6,7]. CFRP composites can also contribute towards bridge structures deteriorated under extreme loading conditions (dynamic and cyclic excitation scenarios). Structures affected under these circumstances are rehabilitated through parallel retrofitting strategies like CFRP [8,9].

Furthermore, many experimental studies have been carried out based on CFRP behavior, and several empirical relationships have been developed so far. Moreover, multiple new studies have also been carried out based on other composites such as GFRP (glass fiber reinforced polymer) by substituting new variants [10]. However, experimental work requires more resources in terms of cost, sophisticated efforts, and time-consuming laboratory tests to develop empirical relationships, thus researchers have been employing artificial intelligence (AI) in multiple engineering fields.

It is vital to mention that multiple AI techniques have been incorporated to explain the solutions based on civil engineering problems amongst various machine learning (ML) algorithms [11,12,13]. Moreover, complex engineering problems can be simplified by utilizing the pattern recognition abilities of common AI algorithms [14,15,16,17,18]. Therefore, typical features of conventional AI techniques, i.e., Neural Network (NN), have been extensively employed by researchers to model various mechanical properties of concrete. These include forecasting light weight based short column concrete compressive strength, predicting compressive capacity for green concrete, high performance concrete, foamed concrete, high strength concrete, lightweight concrete investigation, and prediction of chloride effect in concrete have been studied [19,20,21,22,23,24,25,26]. Moreover, Ghanizadeh et al. [27], Khademi et al. [28], and Reddy [29] utilized NN for predicting the ultimate strength of concrete. Other researchers have utilized NN models to forecast the strength capacity of confined concrete columns and cylinders laminated with FRP, such as Mansouri et al. [30] and [31,32,33,34,35]. Several empirical models of confined concrete for extreme scenarios have been suggested, such as forecasting FRP confined concrete behavior by incorporating typical neutral networks techniques based on limited database, bond strength behavior between FRP composite and concrete, and stress–strain behavior of FRP composites based on ANN [36,37,38,39,40,41,42,43,44,45,46].

It should be noted that even advanced ML algorithms such as ANN can barely perform well, and only for an optimized set of problems, and are therefore perceived as a black-box algorithm. The black box property associated with the algorithm is due to its inadequacy to account for information and physical phenomena of a problem being solved [47]. Thus, corresponding models developed by ANN are perceived as a simple correlation of inputs and outputs, and therefore developed relations will cause new redundancies in models. Such inclusions would result in linear or complex relations based on prespecified base function [48]. Several computational algorithms such as genetic programming (GP) and the model tree are being employed to model concrete properties [49,50,51,52,53,54,55], to elude corresponding redundancies and complexities.

The dominance of these algorithms is the high generalized capability and substantial prediction capacity being achieved through the development of practical mathematical expression. However, AI techniques have been utilized in different civil engineering problems due to enhanced capability and better predictions capacity. Table 1 demonstrates the summary of modeling studies conducted on CFRPs from past researchers based on conventional approaches. The typical aspects of AI techniques have been widely utilized to build an empiric relationship to evaluate the strength capacity of CFRP confined concrete. However, most empirical relationships for CFRP confined concrete in the literature were established by utilizing limited databases, and curve-fit strategies with limited curve fit functions, implying that researchers could not cover all interrelations and combinations of variables to obtain an accurate model.

Furthermore, strength model presented by Fardis [56] is established upon reviewing the model published by Newman [57] and Richart [58], based on FRP confined concrete. All such models have been developed with a small selection of conventional and geometric variations that becomes their flaw, resulting in no guarantee of future relevance. Experimenting with more sample sets and validating the suggested models with comprehensive statistical measures and evolutionary techniques along with proper evaluation of introductory variables through parametric analysis can improve the accuracy of the models. Similar approaches have been adopted by other authors based on conventional tactics and regression analysis techniques. Therefore, substantial study with a wide range of experimental values is necessary to establish a diversified empiric connection, along with an extra thorough strategy.

Therefore, researchers are inquest to collaborate for the assistance of machine learning. These computational intelligence techniques come up with the ability to instinctively discover and improve the systems without being programmed explicitly based on experience. Machine learning emphasizes establishing programs that can access data and manipulate it to learn for themselves. The learning process begins upon examining data, such as experimental data from the past literature, and examining the data patterns to make effective recommendations for the future. The main goal is to allow computers to learn on their own, without the need for human intervention, and attune their actions correspondingly. Therefore, Multi Expression Programming (MEP) as a versatile technique have the ability to encrypt multiple chromosome expressions in a single program exclusively. The best of the designated chromosome is then adopted as the final illustration of the solution [70]. MEP being an advanced variant of gene programming (GP) can speculate results accurately if the complications are unspecified in comparison to other evolutionary algorithms (EAs) [71].

To contemplate the situation, ML program-based algorithm MEP, an advanced tool in AI more realistic than all other traditional and statistical approaches, has been employed to construct a mathematical model for forecasting CFRP-constrained concrete’s compressive strength based on a comprehensive database. Collectively 828 data points have been incorporated in this study which is another outstanding aspect of this work. Thus, the corresponding mechanical characteristic of CFRP confined concrete has been developed by employing MEP, depending upon the majority of affecting and essential factors. A huge database has been compiled from published research and categorized into different sets (train, validate, and test) to assure that the model is adequately trained. Comprehensive statistical analysis and parametric evaluation are performed. The developed model is also compared with existing empirical relationships for CFRP confined concrete to evaluate the credibility of the proposed model to guarantee model generality and validity. The developed empirical relationship can reliably predict confined concrete strength performance, which would be useful in analysis and design considerations for composite concrete structural components made with CFRP. The work is structured as follows: a description of the MEP methodology, an experimentation database collection, modelling methodology, interpretation and discussion of results, parametric and sensitivity analysis, and a summary of the key findings.

## 2. Research Methodology

### 2.1. Multi Expression Programming

**Figure 1:**
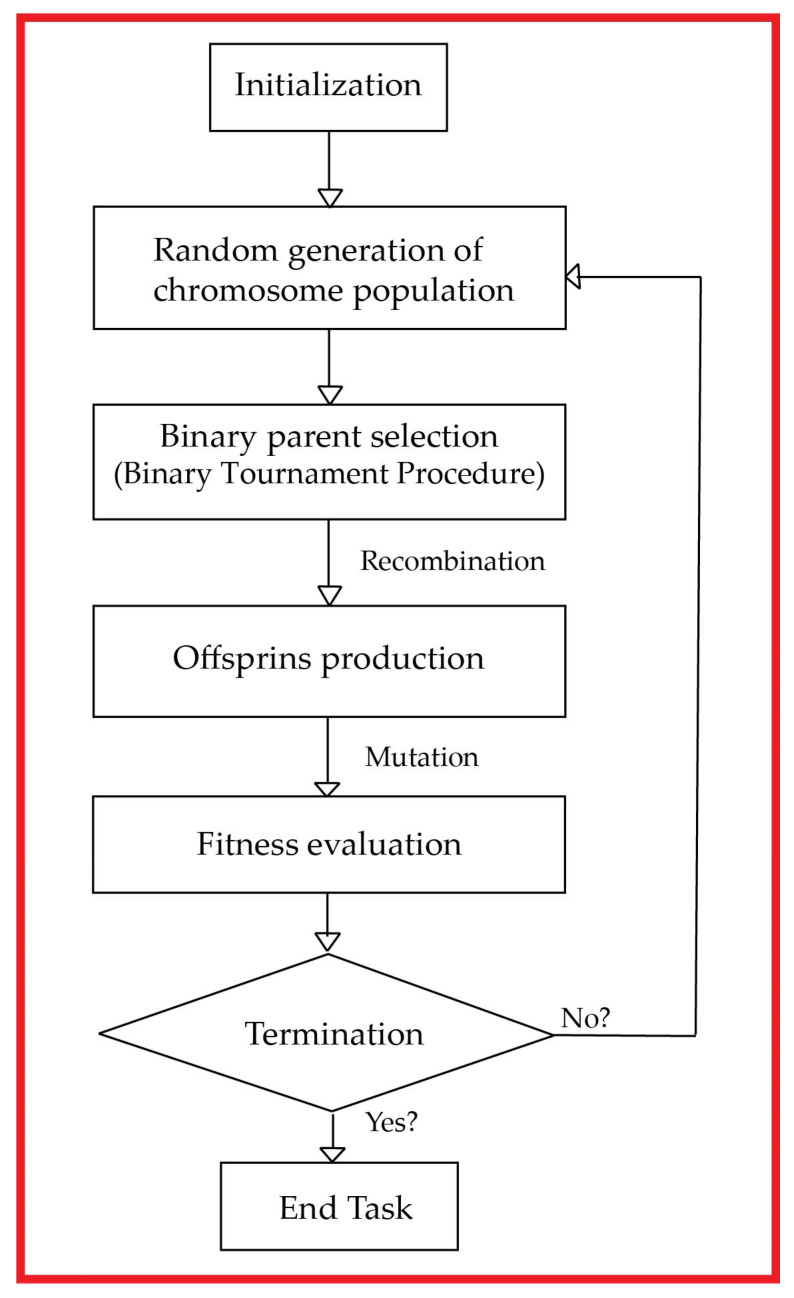
MEP algorithm cyclic representation.

The widespread assignment in most research-based studies is to present a computational model to explicate and forecast specific phenomena or actions. Numerous computational techniques, such as Evolutionary Programming (EP), Multi-Expression Programming (MEP), Genetic Algorithm (GA), and Gene Expression Programming (GEP), were established in this regard to assist these activities [72,73]. The prime focus of AI modeling is to develop feasible and accurate mathematical illustrations to predict the outputs based on pre-specified input parameters. However, the GP-based Darwinian principle idea of natural selection was proposed by [74], which is an evolution of the genetic algorithm (GA). The paramount variance involved in these methods is GA utilization based on fixed binary length strings substituted in GP with nonlinear parse trees. An utmost variant of linear proportionality has already been suggested over the past few years by various EA’s. Independents can also be illustrated as variable-length units as suggested by [75,76] for the MEP case. The simulation of MEP output can be demonstrated as an instruction based on linear strings, where strings are a coalition of mathematical functions and variables. The MEP schematic process is demonstrated below in Figure 1. Moreover, MEP evolution operation begins with the production of chromosomes population randomly. Thus generation begins by utilizing a binary tournament and thereby selecting two parents initially. Reorganization then happens with a cross-over probability, followed by the production of binary offspring and genetic reshuffling of designated parents, and mutation of offspring; replacing begins based on the population of the worst-performing individuals with the optimized one’s. The whole operation being periodic remains continued unless it conflux towards convergence [77].

Most of the presented works done in the last few years put intense consideration upon computational techniques, particularly in Neural Networks and GEP techniques for modeling different problems related to civil engineering. Despite that, MEP has a certain dominance on similar Intelligence techniques. Usually, to analyze the characteristic behavior of concrete, an extensive database is required to forecast the output. Gene Programming utilizes genetic tree crossing based operators, which results in the production of an immense population of processed components, or derivation tree which successively causes an increase in the production time of the model and thus requires extra storage [74]. Furthermore, GP operates as a genotype and phenotype due to the nonlinear structure that makes it strenuous for the algorithmic process to predict reliable mathematical operators for the intended expression [51].

Contrary to this, MEP can identify among the genome and phenome categories due to the involvement of linear variants [78]. In GP the rate of success increases up to a threshold value with the number of genes in chromosomes. Nevertheless, over-fitting is a significant issue and tends to exist in the forecasted properties beyond the limit. That over-fitting limits the model feasibility in the construction sector [26,79,80]. Conversely, MEP is dominant when final expression complications are unspecified, which is a normal practice involved in material science problems. A slight alteration in parameters can alter the results considerably [75]. The capability of MEP makes it possible to code multiple solutions in a single chromosome. In addition, the linear pattern of chromosomes assists the algorithm in exploring wide and vast space in forecasting the target. The dominance MEP has over other computational algorithms makes it able to establish rigorous and robust models for the construction industry. Few studies in the past employ MEP to develop the systematic categorization of soil based on the Atterberg limit (to distinguish the consistency states either plastic limit or liquid limit), gravel occurrence, soil color, the volume of fine-grained particles, and sand percentage [77]. Thus, nonpiecewise models are proposed to aid in determining the degree of soil consolidation [81]. Nevertheless, normal and high strength concrete tangent (E) formulation by [78], models for concrete columns confined with aramid fiber-reinforced polymer (AFRP) [71], soil deformation modulus evaluation [82], formulation of suction caisson uplift capacity [83,84], and CS of Portland cement-based on 28-day strength [85] were among other studies.

However, in the presented study strength model for CFRP confined concrete has been developed by utilizing the MEP approach. The modeling is linked with comprehensive analytical and descriptive studies to assure the validity and efficacy of the created model. The development of credible models will encourage the building industry to use CFRP confined concrete since it eliminates the sophisticated and laborious experimental procedures required to test such an unusual material for construction. The developed approach will help to strengthen infrastructure through retrofitting and rehabilitation, and would also promote viable construction and resource conservation by preventing infrastructure deterioration. Furthermore, the suggested modeling strategy will allow future accurate simulations of similar complicated engineering phenomena.

### 2.2. Experimental Database

For modeling purposes, a thorough database of mechanical and geometrical parameters of CFRP confined concrete was compiled from the publications. The database created in this study is based on earlier experiments. The compiled database provided an extensive dataset of 828 specimens and all critical parameters related to the strength enhancement of concrete enclosed with FRPs. Universal and robust model development was ensured by incorporating all the variable datasets collection. Cube samples were used in some of the investigations to evaluate mechanical characteristics. The cube compression strength was converted to cylinder compression strength by the UNESCO converter coefficients [86] to ensure data conformance and consistency. To determine the probable parameters impacting the characteristics of CFRP confined concrete, thorough literature research and statistical data analysis were performed. Table 2 shows the range and statistical information of the parameters incorporated and employed in the model’s construction. The proposed parameters being included comprised of five inputs and one target component as follows:

Input = {*d*; *h*; *nt*; *f*′*_co_*; *E_FRP_*}

Output = {*f*′*_cc_*}

where *f*′*_cc_* is the confined compressive strength of CFRP. *d*; *h*; *nt*; *f*′*_co_*; and *E_FRP_* are the respective section diameter, the corresponding height of specimen, the CFRP layers thickness, unconfined concrete strength, and finally, the elastic modulus of fibers, appear to be potentially effective parameters in predicting the ultimate load values and thus be utilized as the input parameters to establish the model. Moreover, ***ε_co_*** and ***ε_cc_*** are the corresponding strain values of unconfined concrete and CFRP confined concrete of respective specimens.

**Figure 2:**
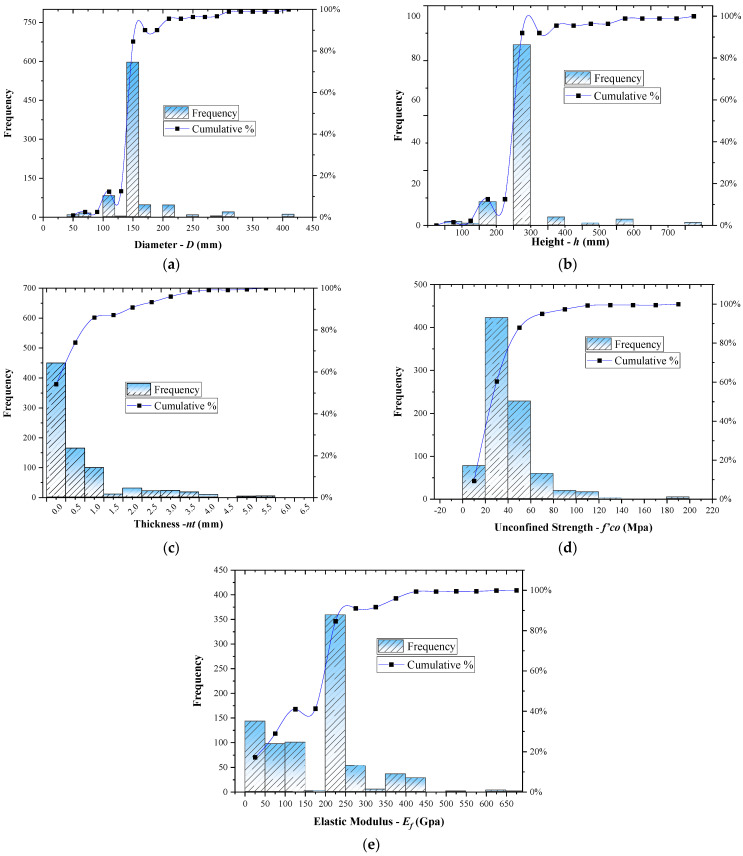
Histogram distribution for: (**a**) *d*, (**b**) *h*, (**c**) *nt*, (**d**) *f*′*_co_*, (**e**) *E*.

The distribution of input parameters has a significant influence on the generalization capacity of the generated model. In Figure 2, data is represented using frequency histograms to depict the distribution of individual variables. As shown in Figure 2, the distributions of the input variables are not consistent, and the frequency rate of the input parameters is relatively high. It is important to remember that if input parameters have a high-frequency rate, we will be able to get a better model. The statistics and ranges of the individual variables used in the model are summarized in Table 2 to make the data more comprehensible. The table depicts the data’s center (mean and median), dispersion (standard deviation and variance), extremes (maxima and minima), and distribution shape (skewness and kurtosis), making data interpretation relatively straightforward. The results reveal that the suggested machine learning models apply to a wide range of input data, boosting their utility.

The multicollinearity problem, which emerges due to the interdependence of input parameters, is a prevalent challenge in the execution of machine learning techniques [49]. It has the potential to raise the strength and endurance of correlations between variables, thus lowering the effectiveness of the produced model. It is recommended that the coefficient of correlation (*R*) between two input parameters remains less than 0.8 [87] to overcome the issue of multicollinearity. *R* is evaluated for all potential input variable combinations, as given in Table 3. The table shows that *R*, whether negatively or positively, is smaller than the stipulated limit (0.8), indicating that there would be no possibility of multicollinearity amongst variables during modeling.

### 2.3. Modeling Parameters

As previously stated, to build a robust and comprehensive model, several fitting parameters for MEP must be specified prior to modeling. These fitting parameters are adopted based on past suggestions using the hit-trial and error procedure [88]. In addition, the population size is specified so that the number of programs incorporated in the population is particularized priorly. Converging a model having a huge size of population would be difficult, sophisticated, and time-consuming. However, once the model’s size is expanded above a certain point, the issue of overfitting may develop. The process was actuated to take into account the number of the subset as 100. Table 4 shows the parameters chosen for the model produced in this study. For the sake of convenience, the function set contains the following mathematical functions of adding, subtracting, multiplying, and divisions, as well as certain trigonometric functions to ensure that the final expressions are robust and accurate. The algorithm’s accuracy level is determined by the number of generations achieved by the algorithm prior to the termination. The best model can be accomplished through the run with as many generations as possible, and consequently, that will result in a model with the fewest anomalies.

Similarly, mutation and crossover rates represent the likelihood of progeny undergoing these genetic procedures. The percentage of cross-over ranges between 50% to 95%. The data was subjected through multiple combinations of modeling configurations, and the optimum combination was adopted based on overall model subjective evaluation, as shown in Table 4. Being an advancement in the modeling procedure, one of the common issues often encountered in AI modeling is data overfitting. A model behaves efficiently with the source data, conversely, it performs poorly with unknown data. Therefore, it has been highly endorsed that the trained model be tested on an unknown or test dataset to avoid conflicts arising from these problems [89,90]. However, the entire database, on the other hand, has been arbitrarily separated into training sets, validation sets, and testing sets.

These training sets and validation sets were treated appropriately during modeling. The verified model is next used to test on the third dataset. However, it is a test set that was not involved in the model’s construction. It must be assured that the data distribution is uniform across all data sets. To maintain consistency in the presented work, 70%, 15%, and 15% of the data are manipulated based on the train, validate, and test sets, respectively. The final models exhibit better performances over all datasets. For this purpose, a commercially accessible computational tool MEPX v1.0 was acquired to employ the MEP algorithm.

The commencement of the algorithm is initiated by producing an initial population of viable alternatives. The mechanism is recursive, thus converges to approach the conclusion with every new generation. In each generation, fitness is at first well appraised inside the solution population. However, in machine learning algorithms, a big concern is model overfitting due to the data training in excess. This overfitting eventually causes an increase in testing error, but training error decreases continuously [91]. Therefore, to cumber the effects caused by the overfitting of the model, the term objective function (*OF*) is introduced in machine learning. This *OF* term is known as a fitness function.

Moreover, from the literature review [49,92] it is proposed that the best model selection should be made based on minimized objective function (*OF*). In the current study, to demonstrate the overall efficacy of the model *OF* is also being assessed for each trained model, as it can consider the effects of *R*, *RMSE*, and the quantity of input data. Hence, the model developed by MEP persistently transforms unless there is no transition recorded in the pre-established fitness function, i.e., *RMSE* or coefficient of determination. Furthermore, the process is repeated until its convergence to achieve an accurate and robust model for these three datasets (training, validation, and testing) by eventually expanding the amount and size of the subpopulation. Finally, the model selection will be made based on the minimum value of *OF*. However, superior performance of some models was indicated for the training set compared to the testing set, which indicates that the model is overfitted and must be countered accordingly. It is to be considered here that the accuracy of the developed model is impacted by the evolution period for the number of generations developed. With the inclusion of each new variable in the programmer in these algorithms, the model is constantly evolving. Therefore, in this research, the generated model was terminated either upon 10,000 generations or when the change in fitness function remained acceptable, i.e., less than 0.1 percent.

Furthermore, an optimal model must satisfy multiple performance indicators, as explained in the following discussion. These performance indicators assess the efficacy of the proposed model by evaluating statistical error and model indicators. These measures include Coefficient of Determination *R*, Relative squared error (*RSE*), Relative root mean square error (*RRMSE*), *RMSE*, Mean absolute error (*MAE*), and Fitness function, performance index (*ρ*). The Equations (1)–(7) represent the relationships for statistical indicators as discussed.

(1) $$R=\frac{\sum_{i-1}^{n}\left(e_{i}-\bar{e}i\right)\left(m_{i-}\bar{m}i\;\right)}{\sqrt{\sum_{i-1}^{n}\left(e_{i}-\bar{e}i\right)²\sum_{i-1}^{n}(m_{i}-\bar{m}i)²}}$$

(2) $$RMSE=\sqrt{\frac{\sum_{i-1}^{n}(e_{i}-m_{i})²}{n}}$$

(3) $$MAE=\frac{\sum_{i-1}^{n}\left|e_{i}-m_{i}\right|}{n}.\;$$

(4) $$RRMSE=\frac{1}{\left|\bar{e}\right|}\sqrt{\sum_{i-1}^{n}(e_{i}-m_{i})²}.\;$$

(5) $$RSE=\frac{\sum_{i-1}^{n}(m_{i}-e_{i})²}{\sum_{i-1}^{n}\left(\bar{e}-e_{i}\right)²}$$

(6) $$\rho =\frac{RRMSE}{1+R}$$

(7) $$OF=\left(\frac{n_{T}-n_{TE}}{n}\right)\rho_{T}+2\left(\frac{n_{TE}}{n}\right)\rho_{TE}.\;$$

Here $e_{i}$. and $m_{i}$, denote the *i*th actual and estimated values respectively, and $\bar{e}i$ and $\bar{m}i$ denote the mean *i*th experimental and average estimated values, respectively, and *n* denotes the total number of observations utilized for modeling. The subscripts *T* and *TE*, respectively, reflect the train and test sets.

Furthermore, several criteria must be observed when evaluating the validity of constructed models. Therefore, as a result, it must meet at least the standards outlined in the literature as follows [93,94,95,96,97,98,99].

1. To exist a correlation between the observed and expected values |*R*| needs to be between 0.2 < |*R*| < 0.8.
2. If |*R*| evaluated to be < 0.2, that depicts a weak correlation among the actual and predicted values.
3. |*R*| has to be larger than 0.8 to maintain a strong correlation between expected and actual values.

Furthermore, a model with a strong *R* and limited predictive errors is considered reliable. In general, the |*R*| value is an important parameter to consider when evaluating a model. Researchers have suggested that *R* be used to assess linear relations between inputs and outputs’ results [22,83]. However, it does not evaluate the overall efficiency of the model due to its impassive behavior towards division or multiplication of output with a constant value. The average magnitude of the errors is calculated using the *RMSE* and *MAE* measures. Each has its own set of implications and restrictions. For instance, in *RMSE*, the average value error is squared before the estimate, resulting in a preference for greater deviations.

In contrast, a large *RMSE* value indicates that such outputs having significant errors are far higher than anticipated and must be minimized. In comparison to *RMSE, MAE* allocates low weightage to larger errors, leading towards less value. Other researchers, such as Despotovic et al. (2016) [100], have recommended that the *RRMSE* value for excellent modeling should be between 0 and 0.10; however, if such calculations were within 0.11 and 0.20, the model is considered good. Other indices, such as ***ρ*** and *OF*, lie between 0 and infinity. However, for the reputation of a good model, ***ρ*** and *OF* must be less than 0.2 [92]. The parameter *OF* has significant importance as it considers the effect of three main statistical parameters involved in training and testing datasets, i.e., *RRMSE*, *R*, and relative percentage.

Furthermore, a lower value of *OF* indicates that a proposed model efficiency is preferably sufficient. The computed OF is preferably close to the criteria stated for a good model in the presented study. As explained earlier, numerous trials were carried out until the model converged to yield the lowest *OF* value. Furthermore, the developed model is externally validated through standards suggested by other scholars, which are presented in Table 5.

## 3. Results and Discussion

### 3.1. Mechanical Properties and Formulation

**Figure 3:**
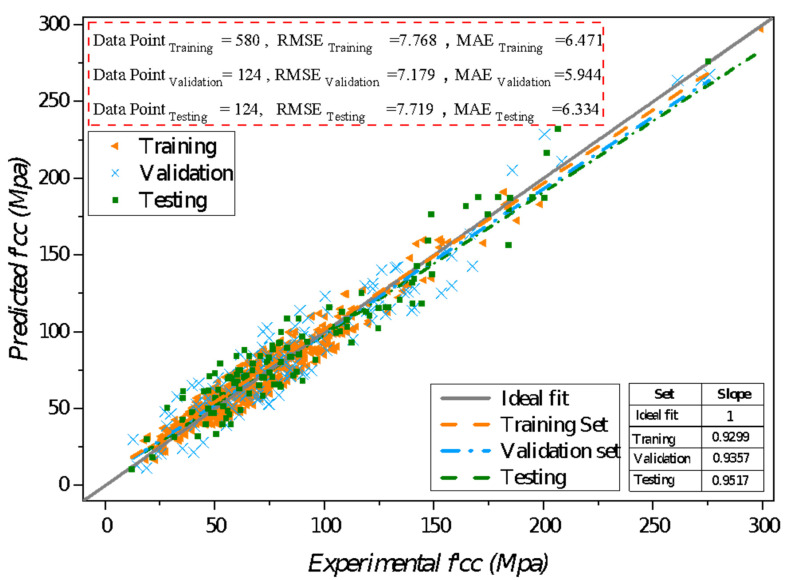
Assessment of predicted *f*′*_co_* vs. experimental output.

The mathematical equations for the computation of CFRP confined concrete strength consist of five input parameters derived by decoding the developed model generated by MEP. The expression for developed mathematical expression is represented by Equations (8)–(11), respectively. In addition, Figure 3 shows the comparison of actual and forecasted *f*′*_cc_* for all data sets: train, validate, and test phase. Furthermore, the slope of the best fit line for all three data sets and the slope for an ideal fit scenario are displayed in the graph. The slope of the best fit line should pass through the origin and approach one for a perfect fit. Figure 3 shows a significant correlation between actual and projected results for all datasets in the created model. The corresponding slopes for train, validate, and test phases are evaluated as 0.9299, 0.9357, and 0.9517, respectively. The results are quite identical and closer to a good fit throughout all sets. This fitting indicates that the model has been efficiently developed and thus has a strong generalization ability, as it behaves well enough on unknown data when forecasting output. The generalization of the established model suggests that the problem of model over-fit has been minimized and reduced on a broad scale. It is also worth noting that the quantity of data points needed for forecasting is eminently reliant on the efficiency and applicability of produced models [103,104,105]. So far, 828 data points have been added into the assembled database for forecasting *f*′*_cc_*, which is another fascinating part of this work; as a result, high precision with few discrepancies has been obtained.

(8) $$\frac{f^{\prime }cc\;}{f^{\prime }co\;}=X+Y+Z$$

where as

(9) X=cos log (2*nt*E)

(10) Y=log (4*nt*E)

(11) Z=tan (cos (log (2*nt*E)

### 3.2. Model Performance and Evaluation:

Before probing into the discussion of model performance, it is equally important to assure the validity of the established model. Therefore, the amount of data utilized in the model development needs to be analyzed, as the model’s accuracy relies on it. The researchers suggested that the proportion of the number of data to the number of input parameters employed for trained and unknown (validation and testing) data be greater than 5 [106]. However, in light of the previous discussion for the training phase, the *f*′*_cc_* model has a ratio of 116. While in the testing stage, the *f*′*cc* model has a ratio of 24.8. As previously stated, numerous statistical measures assess the efficiency of the developed models such as *R*, *RMSE*, *RSE*, *MAE*, *ρ*, *RRMSE*, and *OF*. The values of various statistical measures or Indices for training, validation, and testing sets are demonstrated in Table 6 for the generated *f*′*_cc_* model.

However, it can be deduced from the table that both predicted and experimental values possess a high correlation between them as inferred by *R*-value 0.9948, 0.9950, and 0.9953 for each set of training, validation, and testing, respectively. The values of indicators (*RMSE*, *MAE*, and *RSE*) show high accuracy as they are considerably less and quite close for each dataset which is another sign of the generalized capability of the model. The derived model’s *RMSE* appears to be 7.76, 7.17, and 7.71 for the three sets, respectively. The corresponding *MAE*’s are 6.47, 5.94, and 6.33, respectively, of the developed model. The results inferred that the computed *MAE*’s are less than the *RMSE*, which fulfills the earlier analysis condition. However, the calculation of *RRMSE* infused the significance of the developed model for predicted *f*′*_cc_* as outstanding. Thus computed *RRMSE* indices for each set are less than 0.10, i.e., 0.0045, 0.0098, and 0.0097, respectively.

**Figure 4:**
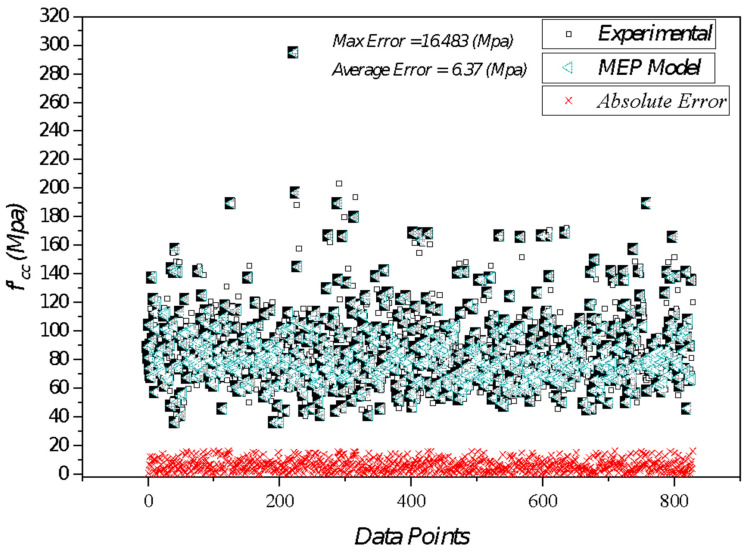
Graphical illustration of absolute error in forecasted and actual output.

Furthermore, findings imply that the evaluated *RRMSE* lies in the range 0 < *RRMSE* < 0.10 for each of three sets of strength models, which shows that the model lies in the excellent range. Moreover, there is also another indication that if the ***ρ*** value remains less than 0.20 for all three sets, the model will be inferred as reliable and proficient for forecasting output. In addition to these statistical measures, another indicator *OF* was incorporated in this study to counter the overfitting problems. Overfitting not only alters the results but is also responsible for forecasting inaccurate outputs. However, the *OF* computed for a developed model is 0.009. This value is exceptionally close to zero, indicating the validity and overall performance of the model and overrule the issues of overfitting by addressing it satisfactorily. The statistics of absolute errors along with respective data points are plotted in Figure 4.

The database utilized in modeling represents that the average error in forecasted values for *f*′*_cc_* is 6.37 Mpa*,* with a maximum error recorded as 16.48 MPa. As quoted initially, the database employed in this study contains 828 points. Among such a huge database, it is worth noting that only 12% of instances have an error greater than 8%. However, the maximum error density obtained based on these data points is not considered high. It is pertinent to mention that approximately 88% of the predictions obtained have errors computed less than 8% for the *f*′*_cc_* model.

**Figure 5:**
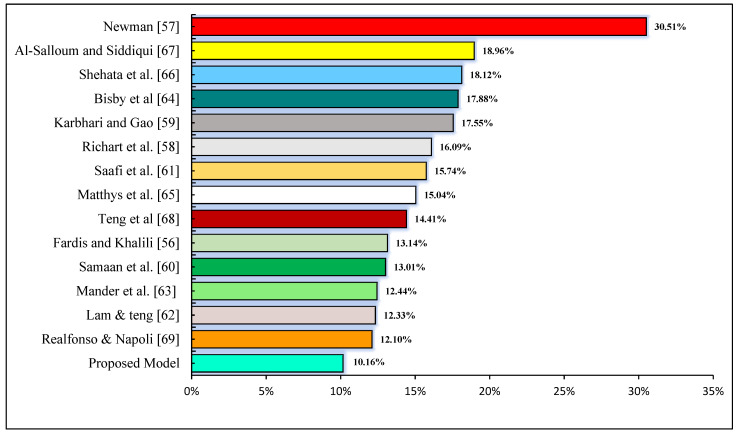
Average absolute error (AEE) of strength enhancement ratio (*f*′*_cc_*/*f*′*_co_*) in predicted model.

Moreover, another measure used to examine the prediction capability of a model is the average absolute error (AAE) or the average percentage discrepancy. In this study, the percentage discrepancy of the developed MEP model from experimental values is only 10.16%. This number is low considering the large database, demonstrating the suggested model’s great accuracy. The suggested model outperforms the existing best-performing models in the literature, as shown in Figure 5, demonstrating the enhanced performance of the forecasted model over the existing best-performing models in the literature. Most of them have employed limited and small databases. Thus, the respective discrepancy in values may indicate failings in credibility and accuracy to forecast the output.

The proposed parameters by [102] have been incorporated in this study to perform an external validation of the established model, as mentioned in Table 7. Therefore, to compute the model resilience and efficacy, it is indispensable that the regression coefficient (*k or k′)* must pass through the origin and be close to 1. In 2008, a researcher [101] proposed a confirming parameter (*R_m_*) to analyze a model’s external reliability. The *R_m_* value must be greater than 0.5 to meet the criterion. However, it is shown in Table 7 that the proposed models meet the external validation criteria, demonstrating that they are credible, resilient, and not just another simple correlation of input and output variables.

### 3.3. Parametric Analysis

An empirical model was proposed and employed for the parametric investigation to evaluate the efficacy of the parameters, which include:

(i) Material properties such as modulus of elasticity (*E*) of FRP, unconfined concrete (*f*′*_co_*) strength;
(ii) Geometric properties such as thickness (*nt*) of FRP composites, and cylinder diameter (*d*).

**Figure 6:**
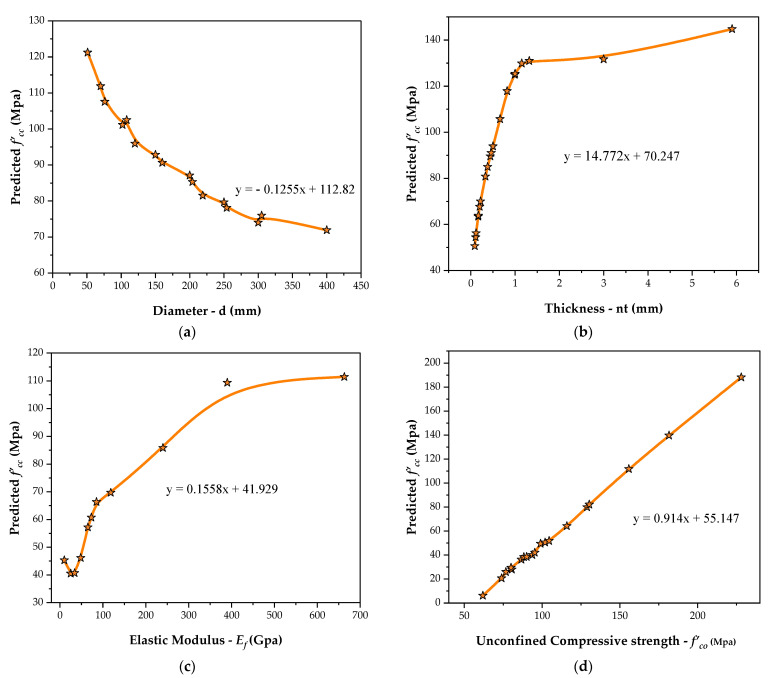
Variations in presented strength model using Input parameters: (**a**) *d,* (**b**) *nt*, (**c**) *E_f_*, (**d**) *f*′*_co_*.

The objective involved in analyzing the behavior of each parameter is to study their effect on the strength of confined concrete. Therefore, it is essential to conduct several analyses before implementing AI-based models to ensure that models have enough resilience and robustness to execute efficiently for all different data combinations. It is to be intimated here that the superiority of the models does not need to be demonstrated through their performance over current data sets, i.e., training, validation, and testing. However, in the presented study, a parametric evaluation technique is being used, as suggested by numerous scholars, to assess if the model has been well-trained and does not simply correlate inputs and outputs. To perform this analysis, an average value of each input parameter needs to be constant and corresponding variance in the output is presented against the variance in one input parameter across its full range. All the input variables being involved in model development go through the same procedure. The parametric analysis results for developed *f*′*_cc_* for each input parameter are shown in Figure 6. These observations are aligned with the experimental study conducted in the past.

#### 3.3.1. Effect of Diameter (*d*)

Concrete specimens with diameters ranging from 50 mm to 400 mm were employed in the parametric analysis, with each increment of 50 mm. The fluctuation of *f*′*_cc_* due to a change in concrete diameter (*d*) is shown in Figure 6a. When the diameter value ‘*d*’ increases from 50 mm, the *f*′*_cc_* for concrete increases at first, then continues to decline up to 400 mm, leaving all other parameters constant. When *d* increases from the initial to the final value, an overall decline of approximately 44% was recorded in *f*′*_cc_* value. In addition, it can be inferred from the graph that there is a gradual decrement observed in the value of *f*′*_cc_* from 118.528 Mpa to 66.977 Mpa up to a final value of *d*. Therefore, it is convenient to envision that the effect on *f*′*_cc_* is significant at smaller diameters.

#### 3.3.2. Effect of Thickness of FRP Layers (*nt*)

The thickness range for FRP layers used in the parametric analysis was 0.09 mm to 5.9 mm, with a 0.5 mm increment. Figure 6b depicts the effect of change on the thickness of FRP layers. The rise in *f*′*_cc_* was outstandingly 147 percent, when *nt* was increased from 0.15 mm to 1 mm, demonstrating that the impact of raising *nt* is more considerable at lower levels. Moreover, for values of *nt* beyond 1 mm to 5.9 mm, there is no significant increment observed in *f*′*_cc_*, i.e., 120.40 Mpa to 140.84 Mpa accounting for a 17% increase in *f*′*_cc_*. It can also be concluded that for higher values of *f*′*_cc_* the effect of raising the thickness of the wraps appears to be less significant. This trend is consistent with prior studies conducted on CFRP confined concrete, as the increase observed in strength between min and max thickness is 188%. However, confined concrete strength (*f*′*_cc_*) generally has a linear relation with the unconfined concrete strength *f*′*_co_*, thus increasing proportionally [107,108]. Apart from this, increasing the thickness of FRP wraps (*nt*) has the same effect as observed in the current study.

#### 3.3.3. Effect of Elastic Modulus of FRP (*E_f_*)

Figure 6c depicts the effect of changing *E_f_*. The *E_f_* values vary from 10 GPa to 663 GPa, with each increment of 50 GPa. When the parameter *E_f_* value exceeds from 10 GPa up to 390 GPa, the *f*′*_cc_* increases by 143 percent. Similarly, when *E_f_* increases beyond 390 Gpa, a slight increase in *f*′*_cc_* can be observed up to 663 Gpa. However, it should be noted that with the increment in *E_f_* from its initial to the final value, an overall improvement in *f*′*_cc_* can be observed. Furthermore, it may be stated that an increase in *E_f_* has a considerable influence on *f*′*_cc_*.

#### 3.3.4. Effect of Concrete Compressive Strength (*f*′*_co_*)

The parametric analysis used several values of unconfined concrete strengths *f*′_*co*_ ranging from 6 MPa to 190 MPa with a 25 MPa increase. Figure 6d shows the variable effect on *f*′_*co*._ When *f*′*_co_* is increased from minimum to maximum while all other parameters remain constant, the forecasted model’s concrete strength rises by 271 percent. In general, the confined concrete strength *f*′*cc* increases linearly by increasing the unconfined concrete strength (*f*′*_co_*).

Therefore, the parametric analysis findings have deduced that *f*′*_cc_* grows as *f*′*_co_* increases, as plotted in Figure 6d. This pattern is also consistent with current design trends. From the foregoing discussion, it can be stated that the constructed MEP model successfully captured the complicated behavior of confined concrete strength and can thus be used widely for future prediction.

## 4. Conclusions

The primary goal of this study was to utilize and examine the capability of the MEP technique in evaluating the compressive strength of concrete confined by CFRP composite. Thus, to present a reliable and efficient model, a large database has been compiled based on extensive research published in the literature. For this reason, a suitable model has been devised to forecast the compressive strength of CFRP confined concrete. The derived findings are in close agreement with the observed/actual data and have a high capacity to anticipate output. Other performance measures such as *RRMSE*, *RMSE*, *R*, *RSE*, and *MAE* were also computed to assess the adequacy and serviceability of the generated models.

The reliable and generalized capability of the established model is verified through ***ρ*** and *OF* indicators which confirms that the problem of overfitting has been managed effectively. The parameter R is exceptionally in the range between (0.9948–0.9953), which depicts the firm relationship among the forecasted and experimental findings for all the datasets incorporated in the study. Higher *R* and lower *MSE*, on the other hand, imply that a high degree of prediction was anticipated for all sets, demonstrating the universality of the suggested model.

The proposed investigation fulfilled the criteria for model external validation satisfactorily. The parametric analysis affirms that the proposed model is capable of forecasting the true behavior for geometrical and mechanical properties of CFRP confined concrete. All the parameters give us an insight that the MEP model in conjunction with validation analysis proved to be an efficient tool to increase the strength capacity. The MEP model was generated by optimizing the training algorithm along with other datasets that assist in decoding the best formulae that are suitable for practicing engineers. The suggested model was also compared to the current strength enhancement ratio (*f*′*_cc_/f′_co_*) published and recommended in the literature by various scholars. Thus, the proposed strength model outperformed all existing models by minimum error projection. The proposed study evaluates the compressive strength of CFRP confined concrete very well by utilizing the developed model. Thus, the corresponding developed empirical relationships have the ability to forecast confined concrete behavior efficiently, which would be useful for analyzing and designing various composite concrete structural members.

## Figures and Tables

**Table 1:** FRP confined concrete strength models proposed by scholars.

Researcher	Year	Developed Model
Richart et al. [58]	1928	$\frac{f_{cc}^{\prime }}{f_{co}^{\prime }}=1+4.1\frac{f_{l}}{f_{co}^{\prime }}$
Newman and Newman [57]	1969	$\frac{f_{cc}^{\prime }}{f_{co}^{\prime }}=1+3.7{\left(\frac{f_{l}}{f_{co}^{\prime }}\right)}^{2}$
Fardis and Khalili [56]	1982	$\frac{f_{cc}^{\prime }}{f_{co}^{\prime }}=1+3.3{\left(\frac{f_{l}}{f_{co}^{\prime }}\right)}^{0.86}$
Karbhari and Gao [59]	1997	$\frac{f_{cc}^{\prime }}{f_{co}^{\prime }}=1+2.1{\left(\frac{f_{l}}{f_{co}^{\prime }}\right)}^{0.87}$
Samaan et al. [60]	1998	$\frac{f_{cc}^{\prime }}{f_{co}^{\prime }}=1+6.0{\frac{\left(f_{l}\right)}{f_{co}^{\prime }}}^{0.70}$
-	-	thus $f_{o}$ = 0.872$f_{co}^{\prime }$ + 0.371$f_{l}$ + 6.258
-	-	E_2_ = 245.61$f_{co}^{\prime }$^0.2^ + 1.3456$\frac{E_{f}t}{D}$
Saafi et al. [61]	1999	$\frac{f_{cc}^{\prime }}{f_{co}^{\prime }}=1+2.2{\left(\frac{f_{l}}{f_{co}^{\prime }}\right)}^{0.84}$
Lam and Teng [62]	2003	$\frac{f_{cc}^{\prime }}{f_{co}^{\prime }}=1+3.3\frac{f_{l}}{f_{co}^{\prime }}$
Mander et al. [63]	2005	$\frac{f_{cc}^{\prime }}{f_{co}^{\prime }}=\sqrt[{2.254}]{1+7.94\frac{f_{l}}{f_{co}^{\prime }}}-2\frac{f_{l}}{f_{co}^{\prime }}-1.254$
Bisby et al. [64]	2005	$\frac{f_{cc}^{\prime }}{f_{co}^{\prime }}=1+2.425\frac{f_{l}}{f_{co}^{\prime }}$
Matthys et al. [65]	2006	$\frac{f_{cc}^{\prime }}{f_{co}^{\prime }}=1+2.3{\left(\frac{f_{l}}{f_{co}^{\prime }}\right)}^{0.85}$
Shehata et al. [66]	2007	$\frac{f_{cc}^{\prime }}{f_{\mathrm{co}}}=1+2.4\frac{f_{l}}{f_{co}^{\prime }}$
Al-Salloum and Siddiqui [67]	2009	$\frac{f_{cc}^{\prime }}{f_{co}^{\prime }}=1+2.312\frac{f_{l}}{f_{co}^{\prime }}$
Teng et al. [68]	2009	$\frac{f_{cc}^{\prime }}{f_{co}^{\prime }}=1+3.5\left(\rho_{k}-0.01\right)\rho_{\varepsilon }$
Realfonso and Napoli [69]	2011	$\frac{f_{cc}^{\prime }}{f_{co}^{\prime }}=1+3.57\frac{f_{l}}{f_{co}^{\prime }}$

**Table 2:** Statistical data about the variables employed in the model.

Parameters	*d*	*h*	*nt*	*E*	*f*′*_co_*	*f*′*_cc_*	*ε_co_*	*ε_cc_*
-	(mm)	(mm)	(mm)	(Gpa)	(Mpa)	(Mpa)	(%)	(%)
Mean	154.62	307.88	0.82	182.52	40.56	74.58	0.26	1.53
Median	152	304	0.38	230	36.3	66.78	0.24	1.35
Mode	150	300	0.33	230	24.5	63	0.24	0.95
Sample Variance	1927.85	7552.62	0.992	12,592.78	469.98	1125.324	0.0155	0.716
Skewness	2.71	2.85	2.355	0.4467	2.603	2.05988	7.428	0.957
Standard Error	1.53	3.02	0.03	3.899	0.75	1.17	0.004	0.031
Kurtosis	12.59	13.48	5.784	0.3353	11.696	8.54763	60.888	0.658
Standard Deviation	43.907	86.91	0.996	112.218	21.68	33.546	0.1246	0.846
Minimum	51	102	0.09	10	6.2	17.8	0.1676	0.083
Maximum	406	812	5.9	663	188.2	302.2	1.53	4.62
Range	355	710	5.81	653	182	284.4	1.3624	4.537

**Table 3:** The coefficient of correlation among different input parameters.

-	*d*	*h*	*t*	*E*	*f*′*_co_*
*d*	1	0.99	0.02	0.07	−0.09
*h*	0.99	1	0.02	0.07	−0.09
*t*	0.02	0.02	1	−0.49	0.19
*E*	0.07	0.07	−0.49	1	−0.10
*f*′*_co_*	−0.09	−0.09	0.19	−0.10	1

**Table 4:** Parameter configuration for MEP algorithm.

Parameters	Settings
Size of subpopulations	150
Number of subpopulation	100
Mathematical operators	+, −, ×, ÷, *Cosθ*, *Sinθ, tanθ*
Crossover probability	0.92
Mutation probability	0.01
Variables	0.5
Operators	0.5
Number of generations	10,000

**Table 5:** Statistical measures of the generated models for external validation.

S. No.	Equation	Condition	Suggested by
1	$R_{m}=R^{2}\times \left(1-\sqrt{\left|R^{2}-R_{o}^{2}\right|}\right)$	$R_{m}>0.5$	(Roy and Roy, 2008) [101]
-	where $R_{o}^{2}=1-\frac{\sum_{i=1}^{n}{\left(m_{i}-e_{i}^{o}\right)}^{2}}{\sum_{i=1}^{n}{\left(m_{i}-{\underline{\bar{m}}}_{i}^{o}\right)}^{2}},e_{i}^{o}=k\times m_{i}$	$R_{o}^{2}\;≅1$	-
-	$R\prime_{o}^{2}=1-\frac{\sum_{i=1}^{n}{\left(e_{i}-m_{i}^{o}\right)}^{2}}{\sum_{i=1}^{n}{\left(e_{i}-{\underline{\bar{e}}}_{i}^{o}\right)}^{2}},m_{i}^{o}=k\prime \times e_{i}$	$R\prime_{o}^{2}\;≅1$	-
2	$k=\frac{\sum_{i=1}^{n}\left(e_{i}\times m_{i}\right)}{\sum_{i=1}^{n}e_{i}^{2}}$	0.85<k<1.15	(Golbraikh and Tropsha, 2002) [102]
3	$k\prime =\frac{\sum_{i=1}^{n}\left(e_{i}\times m_{i}\right)}{\sum_{i=1}^{n}m_{i}^{2}}$	0.85<k′<1.15	[102]

**Table 6:** Statistical indices for training, validation, and testing sets of the established models.

-	*RMSE*	*RSE*	*MAE*	*RRMSE*	*R*	*ρ*	*OF*
Training	7.768321	0.010346	6.471356	0.005	0.9948	0.002291	0.009156
Validation	7.17975	0.009859	5.944429	0.009	0.9950	0.004578	-
Testing	7.719133	0.009733	6.33431	0.010	0.9953	0.004921	-
Database	7.6756	0.0102	6.3719	0.004	0.9949	0.00189	-

**Table 7:** Statistical indices for external validation of generated model.

Sr. No.	Parameters	Sets			Database
		Training	Validation	Testing	
1	*k*	0.991410	0.993896	1.011315	0.994896
2	*k′*	0.998249	0.981181	0.979612	0.994932
3	*R_m_*	0.889943	0.892999	0.896258	0.891061
4	$R_{{}^{\circ}}^{2}$	0.999809	0.999783	0.999783	0.999802
5	$R_{{}^{\circ}}^{\prime 2}$	0.999823	0.999796	0.999794	0.999816

## Data Availability

The data used in this research was collected from published literature.
